# Dyads in Dementia Care: An International Perspective on Research Challenges and Opportunities

**DOI:** 10.1093/geroni/igaa057.2731

**Published:** 2020-12-16

**Authors:** Marie Boltz, Karin Wolf-Ostermann, Katie Maslow

## Abstract

Dementia poses a societal challenge that is life-changing not only for persons with dementia (PWD) but also for family members and friends (informal carers) directly involved in the care arrangement. Informal carers (IC) have typically poorer outcomes in terms of well-being, quality of life (QoL), health status, and use of health care resources. Dyads of PWD and IC living with dementia are characterized by strong reciprocal relationships and complex living contexts. Therefore, research should investigate home based dementia caregiving from a dyadic perspective to yield interventions that support the PWD, the IC, and the unit as a whole. However, it is an ongoing challenge to investigate dyadic needs and preferences in daily practice and develop effective interventions. Challenges are related to incomplete understanding of dyadic characteristics, attitudes and beliefs within the dyad, as well as how to adapt research approach to engage and retain the dyad in research. This international symposium will therefore address these issues. The first presentation will describe a typology of dementia care dyad characteristics and needs in Germany. The second presentation will examine the challenges and opportunities associated with recruiting and retaining dementia dyads. The third presentation will explore ethical challenges posed in communication with dyads and possible solutions for the researcher. The final presentation reports on the Meeting Centre Support Program as an example of an effective psychosocial intervention employing research strategies that transcend cultural barriers. Our discussant, Katie Maslow, will synthesize the presentations and lead a discussion of future directions for policy and practice.

